# Food for IDEAL thought: redesigning junior journal clubs to enhance surgical innovation

**DOI:** 10.1111/ans.17994

**Published:** 2022-10-11

**Authors:** Joshua G. Kovoor, Brandon Stretton, Joseph N. Hewitt, Aashray K. Gupta, Christopher D. Ovenden, Stephen Bacchi, Jonathan Henry W. Jacobsen, Guy J. Maddern

**Affiliations:** ^1^ Information in Surgery Journal Club Adelaide South Australia Australia

*“Knowledge,” in the sense of information, means the working capital, the indispensable resources, of further inquiry; of finding out, or learning, more things*.

John Dewey, *Democracy and Education* (1916).^1^

## Philosophy of evidence‐based surgery

Evidence‐based surgery (EBS) comprises continuous critical evaluation and proactive improvement of surgical care via the scientific method. Novel information must be sought and understood, and scientifically‐validated methods of improving practice integrated safely. For individuals to practice EBS, they require knowledge of the underlying philosophy and the self‐confidence and creativity to innovate reliably.^2^ The stages in the development and assessment of surgical innovations are characterized in the descriptive model delineating stages of innovation, development, exploration, assessment and long‐term study (the IDEAL model), developed by the Balliol Collaboration.^3^ Effective EBS education should involve teaching students to critically appraise and use information to reliably think, learn and succeed independently^1^ throughout their surgical careers. A scalable, low‐cost model that teaches the underlying philosophy of EBS while increasing the research activity of junior participants may enhance the future practice of EBS. This article aims to introduce journal clubs as a means to promote EBS at this early stage.

## Journal clubs and information flow

Journal clubs have been utilized across healthcare disciplines since the 1800s, traditionally involving a group regularly meeting to discuss scientific literature.^4^ They encourage participants to read and critically appraise peer‐reviewed articles relevant to clinical practice. In surgical disciplines, journal clubs can improve resident knowledge of evidence‐based processes, thus potentially facilitating EBS.^5^ One benefit is that they are typically held within academic institutions at no individual expense apart from a time investment. More recently, some have been successfully facilitated using the internet and social media. However, strong evidence supports in‐person journal club models being significantly more effective in teaching evidence‐based surgical principles that those delivered online.^6^ Successful journal clubs can produce academic communities with a multi‐directional flow of information and ideas. For junior participants, translating this information flow to measurable research output could foster future surgical innovation.^7^, ^8^ Similarly, the provision of effective education on EBS within meetings could enhance future EBS by attendees.^8^, ^9^ Most current journal clubs cater for specialists and specialists‐in‐training. For example, in 2004 Toedter *et al*. outlined the implementation of journal clubs within an assignment‐based collaborative training program including attending surgeons, medical librarians and research coordinators aiming to teach surgical trainees evidence‐based surgical principles.^10^ The vacuum of journal clubs catering to juniors beginning their surgical careers remains to be filled. The two primary purposes of a journal club to fill this need would be to improve both engagement with EBS and research publications.

## IDEAL as an educational framework

The latest IDEAL Recommendations provides a model by which EBS philosophy can be conceptualized and impactful surgical innovation guided.^2^ Teaching the IDEAL framework can give students a foundation upon which they can become future leaders in improving EBS. Providing this education within junior journal club meetings alongside standard literature discourse may instil EBS philosophy for attendees with regular reinforcement for long‐term retention. Adding this component would also improve the evidence‐based focus of journal clubs that follow a traditional model of operation. To improve sustainability, meetings could be themed around the IDEAL stages of surgical innovation. Six successive journal club meetings corresponding to the six components of the latest IDEAL framework^2^ may provide robust understanding, particularly if conducted weekly or fortnightly. The core of each meeting will comprise discussion relating to one stage, so that across six meetings all six stages will be covered. A complementary curriculum would include educational materials, presentations and discussions of discrete articles.

## Increasing junior surgical research productivity

Current research productivity of graduating USA general surgery chief residents is poor, as the reported median number of publications is three and median h‐index one.^11^ This is not a geographically confined issue, as among Australian fellows of the Royal Australasian College of Surgeons the median h‐index is two.^12^ This low level of research productivity amongst experienced and well‐resourced members of the surgical community may not only indicate a lack of understanding of methods of publishing, but also a lack of desire to innovate. Young surgical researchers must be supported^7^ so that potential advancements to clinical care are not lost.^8^

With their high levels of information flow, journal clubs present a setting for substantially increasing research engagement at the junior level that is currently underutilized. They give attendees a forum to discuss past or current ideas, and may stimulate the creation of new ones. To act as a mechanism for increasing junior surgical research productivity, journal clubs must be open to allowing members to present novel ideas with the confidence that those receiving them will be supportive and well‐intentioned. Leaders within journal clubs must understand the academic processes of designing, conducting and publishing research and be willing to share their knowledge with participants who do not. A journal club may stimulate junior individuals to undertake low‐risk study designs that can provide a safe, low‐cost and accessible foundation for more impactful future work.^8^ Even if not directly involved, senior academic surgeons open to supervising such projects can be identified. Where possible in the same university or health network, junior journal clubs should have links to senior journal clubs to maintain optimal academic development of individual attendees and the group as a whole. Links with other programs that promote research, such as mentorship from senior staff, research institutions and academic research societies, should be solidified where possible. Attendee research output can be increased through this mechanism, and to monitor rate of growth a count of group publications and h‐indexes should be kept.^7^ No single scientometric metric is perfect in provided an optimal representation of an individual's academic output, and all surgeons should be encouraged to have a strong academic interest. When attempting to gauge innovative productivity or potential, particularly at the junior level, it is important to remember that research engagement is wider than just publishing scientific articles. All forms of surgical innovation, regardless of whether they result in publications, should be encouraged within the academic community created by junior journal clubs. While increasing research productivity is crucial, it is not the only output of a successful junior journal club.

## Principles of redesign

**Fig. 1:**
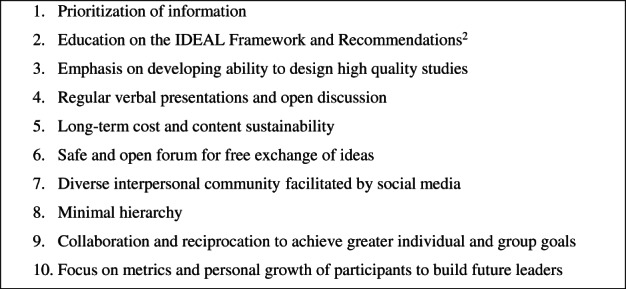
A junior journal club model to enhance evidence‐based surgical innovation.

Ten principles by which junior journal clubs could be redesigned to enhance evidence‐based surgical innovation are outlined in Fig. 1. Primarily, information (synonymous with evidence or data) should be prioritized. The journal club should be structured to optimize information inflow from the surgical community to participants, multidirectional flow between participants within meetings, and outflow from participants back to the surgical community. This may manifest a participant monitoring new publications and communicating these findings to the rest of the group; subsequently sparking an idea for a study that is conducted by the group with supervisor guidance. Education should be provided on the latest IDEAL Framework and Recommendations^2^ to provide attendees with a conceptual foundation for future EBS. This education should focus on themes and fostering discussion rather than purely didactic content with short half‐life. This may promote long‐term sustainability as an educational model. There should be an emphasis on developing the ability to design high‐quality studies of strong design. Developing individual presentation and communication skills should be prioritized, with each session including verbal presentations and open discussion. Meetings should ideally be held in a location of no financial cost to the individual participants, such as an academic institution or conference room within a hospital. Further, given that the schedules of attendees are likely to be busy, flexibility in scheduling, regularity in sessions, and no consequences for non‐attendance should be essential.

Each journal club meeting should present a safe and open forum for the free exchange of ideas. The environment that is created should be one conducive to innovation driven by the individuals involved. The journal club itself should be accessible to anyone ranging from medical students to those formally enrolled in surgical training. This ensures that future surgeons receive EBS education and access to a mechanism by which their ideas can become realized at the stage of their career with the greatest long‐term impact. Open access will also ensure an interpersonal community that is diverse in ideas and demographics such as race, sexual orientation and gender. The diversity in ideas presented and people involved, in addition to the lack of barriers to entry, should allow sustainability and longevity of these journal clubs. Communication between journal club meetings could be facilitated by social media and junior doctor societies within hospitals and universities. Where there are opportunities for virtual hosting, and circumstances where utilizing telehealth modalities^13^ may benefit wider audiences, these should be explored. Minimal hierarchy should be present to optimize inclusivity and individual sharing of ideas. Shared decision‐making should be prioritized at all times.^14^ A leader should be designated to coordinate and lead meetings, but should not be given a title and should prioritize the interests of the group. This responsibility could potentially be rotated within the group once the journal club is sufficiently established. Reduced hierarchy should foster collaboration and reciprocation, facilitating the realization of individual and group ideas. This environment could also be used to nurture junior doctor well‐being, that can address issues of burnout, attrition and the emotional and mental impact of surgical training.^15^ Personal growth of participants should be emphasized and nurtured by the journal club community. Individual achievement should be encouraged, facilitated and celebrated appropriately. Regardless of their characteristics, each individual should be encouraged towards becoming future leaders of EBS. Recognizing and characterizing individual growth will be informed by metrics of research productivity,^7^ which will be emphasized by the group as a whole.

## Future potential

With large amounts of less reliable information entering the surgical literature after COVID‐19, widespread adherence to EBS philosophy is required to facilitate consensus adaptation.^16^ With recent global geopolitical developments, the effects of COVID‐19 may last longer than first thought.^17^ The paucity of research and surgical innovation amongst the senior surgical community^11^, ^12^ highlights that a mechanism of fostering surgical innovation at the junior level is needed. The described junior surgical journal club model addresses these needs at minimal cost or risk, so is universally implementable. It also advocates for a collaborative approach within the academic community, which is a method of fostering EBS on a large scale. While there is a concern that making a journal club of this nature could be too multifaceted, however, the celebration and facilitation of individual growth allow streamlined relevance to anyone in attendance The subsequent benefits that may be conferred to surgical care are immeasurable.

## Author contributions

**Joshua G. Kovoor:** Conceptualization; investigation; writing – original draft; writing – review and editing. **Brandon Stretton:** Conceptualization; investigation; writing – review and editing. **Joseph N. Hewitt:** Conceptualization; investigation; writing – review and editing. **Aashray K. Gupta:** Conceptualization; investigation; writing – review and editing. **Christopher D. Ovenden:** Conceptualization; investigation; writing – review and editing. **Stephen Bacchi:** Conceptualization; investigation; writing – review and editing. **Jonathan Henry W. Jacobsen:** Conceptualization; investigation; writing – review and editing. **Guy J. Maddern:** Conceptualization; investigation; supervision; writing – review and editing.
